# Clinical predictors of Alzheimer's disease‐like brain atrophy in individuals with memory complaints

**DOI:** 10.1002/brb3.3506

**Published:** 2024-04-30

**Authors:** Ahmet Alp Karakasli, Esra Ozkan, Melike Karacam Dogan, Duygu Cap, Ayca Karaosmanoglu, Sevilay Karahan, Nabi Zorlu, Esen Saka, Yavuz Ayhan

**Affiliations:** ^1^ Department of Psychiatry Hitit University Faculty of Medicine Çorum Turkey; ^2^ Research Center for Translational Medicine, Koç University İstanbul Turkey; ^3^ Department of Psychiatry Eregli State Hospital Zonguldak Turkey; ^4^ Department of Psychology Ufuk University Ankara Turkey; ^5^ Department of Radiology Hacettepe University Faculty of Medicine Ankara Turkey; ^6^ Department of Biostatistics Hacettepe University Faculty of Medicine Ankara Turkey; ^7^ Department of Psychiatry İzmir Katip Çelebi University Faculty of Medicine İzmir Turkey; ^8^ Department of Neurology Hacettepe University Faculty of Medicine Ankara Turkey; ^9^ Department of Psychiatry Hacettepe University Faculty of Medicine Ankara Turkey

**Keywords:** Alzheimer, cortical thickness, imaging biomarkers, subjective cognitive decline

## Abstract

**Objectives:**

The definition and assessment methods for subjective cognitive decline (SCD) vary among studies. We aimed to investigate which features or assessment methods of SCD best predict Alzheimer's disease (AD)‐related structural atrophy patterns.

**Methods:**

We assessed 104 individuals aged 55+ with memory complaints but normal cognitive screening. Our research questions were as follows: To improve the prediction of AD related morphological changes, (1) Would the use of a standardized cognitive screening scale be beneficial? (2) Is conducting a thorough neuropsychological evaluation necessary instead of relying solely on cognitive screening tests? (3) Should we apply SCD‐plus research criteria, and if so, which criterion would be the most effective? (4) Is it necessary to consider medical and psychiatric comorbidities, vitamin deficiencies, vascular burden on MRI, and family history? We utilized Freesurfer to analyze cortical thickness and regional brain volume meta‐scores linked to AD or predicting its development. We employed multiple linear regression models for each variable, with morphology as the dependent variable.

**Results:**

AD‐like morphology was associated with subjective complaints in males, individuals with advanced age, and higher education. Later age of onset for complaints, complaints specifically related to memory, excessive deep white matter vascular lesions, and using medications that have negative implications for cognitive health (according to the Beers criteria) were predictive of AD‐related morphology. The subjective cognitive memory questionnaire scores were found to be a better predictor of reduced volumes than a single‐question assessment. It is important to note that not all SCD‐plus criteria were evaluated in this study, particularly the APOE genotype, amyloid, and tau status, due to resource limitations.

**Conclusions:**

The detection of AD‐related structural changes is impacted by demographics and assessment methods. Standardizing SCD assessment methods can enhance predictive accuracy.

## Graphical abstract

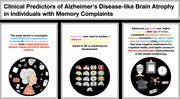

## 1 INTRODUCTION

Alzheimer's disease (AD) is the most neurodegenerative disorder and the most common cause of dementia, typically characterized by progressive impairments in memory and learning that eventually disrupt basic daily activities (Alzheimer's Association, 2023). AD and other dementias affect over 55 million people worldwide and are projected to affect 139 million people by 2050; and the global cost of dementia is estimated to be over 1,3 trillion USD (World Health Organization, 2021). Disease‐modifying treatment options for AD are imminent thus it is necessary to develop effective methods for early detection of AD (Mintun et al., 2021; Sims et al., 2023; Swanson et al., 2021; Van Dyck et al., 2023).

AD pathological changes begin to develop almost 20 years before cognitive symptoms appear (Jack et al., 2011, 2018). Cognitively unimpaired individuals who have abnormal AD biomarkers are considered to be in the preclinical stage of AD. In the late phase of this stage, some individuals may notice and be concerned about subtle changes in their cognitive abilities (Jessen et al., 2020). The self‐experienced decline in cognitive capacity without objective cognitive impairment is defined as subjective cognitive decline (SCD) (Jessen et al., 2014). SCD could serve as a target stage for early intervention as it is the earliest identifiable clinical manifestation of AD continuum.

The definition of SCD is not specific to preclinical AD. In a cognitively unimpaired individual, subjective cognitive complaints can also arise from normal aging, personality traits, psychiatric, neurological, or other medical disorders, substance use, and medications. To increase the predictive validity of SCD in AD, Jessen and colleagues proposed SCD‐plus criteria:

1. Subjective decline in memory, rather than other domains of cognition;
2. Onset within the last 5 years,
3. Age at onset over 60 years,
4. Concerns associated with SCD,
5. Feeling of worse performance than others,

If available or possible to obtain in the respective study:

1. Confirmation of cognitive decline by an informant,
2. Presence of the APOE ε4 genotype and,
3. Biomarker evidence for AD (Jessen et al., 2014). The presence of psychiatric or neurologic disease, a medical disorder, medication, or substance use was considered exclusionary.

The assessment and implementation criteria of SCD vary among studies, with some evaluating complaints with a single question and others using scales (Rabin et al., 2015). The variation in assessment approaches might impact the accuracy with which SCD can predict the AD‐like structural changes and conversion to MCI. For example, Morrison et al. (2022) found that defining SCD not merely as expressing worry about memory/thinking abilities but through assessing cognitive complaints with the Everyday Cognition Scale was found to be associated with increased atrophy in the superior temporal regions. Additionally, the impact of cerebrovascular pathology, as evidenced by overall and regional white matter hyperintensities on MRI scans, differences between individuals with or without SCD were significantly dependent on the method used to operationalize SCD as expressing worry about memory or using valid scales (Morrison et al., 2023). Similarly, a 2018 study by van Harten et al. (2018) demonstrated that combining consistent self‐reported cognitive decline with worry about memory significantly improves the prediction of MCI risk.

In addition to the heterogeneity in the methods of assessing complaints, there is no clear boundary between normal cognition and cognitive impairment when measuring cognitive performance objectively (Molinuevo et al., 2017). In an individual with subjective cognitive complaints, the objective indication of cognitive decline is often determined by using either brief cognitive screening instruments or more extended psychometric tests. Although Jessen and colleagues highlight the limited diagnostic accuracy of brief screening tests, such as the Mini–Mental State Examination or the Montreal Cognitive Assessment, they advocate for the use of comprehensive neuropsychological test (NPT) batteries that evaluate multiple cognitive domains and for which normative data are available. However, there is insufficient data to determine whether utilizing detailed neuropsychological assessments over cognitive screening tests enhances the predictive capability of SCD (Jessen et al., 2020).

Psychiatric disorders, such as depressive or anxiety disorders, are identified as exclusion criteria by SCD‐I because they can independently cause subjective cognitive complaints. On the other hand, psychiatric comorbidity is well‐known in predicting dementia, yet the impact of excluding these disorders on the association between SCD and preclinical AD remains understudied (Desai et al., 2021; Liew, 2020).

The variability in the assessment of SCD results in disparate outcomes (Ávila‐Villanueva et al., 2018; Eckerström et al., 2017; Fernández‐Blázquez et al., 2016; Mazzeo et al., 2020). Changes in the definition of SCD affect its predictive value (Pike et al., 2022), necessitating refinement and validation of SCD‐plus and exclusion criteria.

While evaluating individuals with cognitive complaints, MRI is a valuable tool to assess the topographic distribution of cortical and subcortical atrophy. AD and other neurodegenerative disorders have a focal onset and a predictable atrophy pattern that is helpful in diagnosis. In early phases of the disease, inconspicuous atrophy may precede overt clinical symptoms. Understanding which factors make individuals with cognitive complaints available prone to AD will help earlier detection of these individuals.

Early detection of AD pathology is possible via evaluation of CSF markers or PET imaging (Jack et al., 2018). More convenient blood‐based biomarkers are also emerging (Alcolea et al., 2023). Currently, widespread use of these biomarkers is not possible globally. Identifying the clinical risk factors that predict AD‐like atrophy can help select those individuals that may undergo further investigation. In addition, at‐risk individuals may be candidates for noninvasive prevention strategies such as cognitive training and rehabilitation and lifestyle‐based interventions (Ngandu et al., 2015).

In this study, we aimed to find which clinical feature of SCD is associated with AD‐related atrophy. Specifically, we evaluated the value of using:

1. a valid scale rather than a single‐question assessment,
2. detailed NPT rather than a screening‐based diagnosis,
3. SCD‐plus criteria rather than a single‐question assessment,
4. exclusionary criteria, family history, and vascular burden in predicting AD‐related atrophy within SCD diagnosis.

## 2 MATERIALS AND METHODS

### 2.1 Participants

The participants in this study were drawn from two sources: a field study database and individuals referred to our clinics with memory complaints. The details of our community study are explained in a reference (Ayhan et al., 2018). All participants gave informed consent to participate in the study. The study protocol was approved by the Hacettepe University Non‐Interventional Clinical Research Ethics Board (GO 18/910‐23).

We included participants who met the basic definition of SCD, which required them to (i) be over the age of 55, (ii) have memory‐related complaints, and (iii) score above 1.5 standard deviations (SD) of age‐, sex‐, and education‐adjusted normal performance on the Modified Mini–Mental Test (3MS). Participants who had developmental or intellectual disabilities, sensory impairments that could interfere with neuropsychological evaluation, chronic neurological and psychiatric diseases that may impair cognition and affect morphology (such as schizophrenia, bipolar disorder, or demyelinating diseases), or a contraindication to MRI scanning were excluded from the study.

### 2.2 Study design

We conducted a clinical evaluation after obtaining written informed consent from all participants. We collected information on sociodemographic factors as well as the characteristics and history of cognitive complaints, comorbid diseases, current medications and supplements, and family histories of blood relatives up to the third generation. All psychiatric evaluations were performed by psychiatrists. Furthermore, we administered NPT batteries, blood tests related to cognitive impairment, and conducted brain magnetic resonance imaging.

### 2.3 Cognitive complaints

The presence of memory complaints was determined with the first question of subjective memory complaints questionnaires (SMCQ): “Do you think that you have a memory problem?” To evaluate memory complaints with a valid scale, SMCQ‐Turkish was used as a continuous variable (Kizil Özel et al., 2013; Youn et al., 2009).

### 2.4 SCD‐plus criteria

The SCD‐plus criteria (Van Dyck et al., 2023) were evaluated as follows: (1) “Do you think that you have a memory problem?”, (2) “When did your memory start to become worse?”, (4) “Does your memory decline worry you?”, and (5) “Do you think that your memory is poorer than that of other people of a similar age?”. The third criterion (age at onset over 60 years) was calculated from the first and second questions. The sixth criterion (confirmation with the informant) was assessed with the primary caregiver when possible. We were not able to check the APOE genotype, amyloid status, and tau status in our analysis due to the unavailability of these methods.

Subjective complaints in non‐amnestic domains (visuospatial, language, and attention) were systematically evaluated using a specialized questionnaire developed by the authors. Although this questionnaire has not undergone formal validation, it was meticulously designed based on existing literature and expert consultation to ensure comprehensive coverage of relevant symptoms. Visuospatial, language, and attention complaints were not treated as separate independent variables. Instead, we aggregated complaints across these non‐amnestic domains into a single categorical variable labeled “Non‐amnestic complaints.” If participants reported complaints in any of these domains—visuospatial, language, or attention—they were coded as “1” for *non‐amnestic complaints*, otherwise “0.” The questionnaire, including the specific questions asked, is provided in Table S1.

### 2.5 Comorbidities and medications

Each participant was evaluated by a study physician and the medical history was obtained including comorbid conditions and the medications. We grouped the comorbidities into three categories: psychiatric, vascular, and other. Psychiatric comorbidity included the individuals with clinical depression or anxiety disorder based on the ICD‐10. Vascular comorbidity was identified as the composite score determined based on the presence of hyperlipidemia, hypertension, diabetes mellitus, coronary artery disease, or previous cerebrovascular disease, with each condition given one point. Other comorbidities group included individual diseases, including chronic illnesses such as migraine, previous head trauma, chronic kidney, pulmonary, liver disease, or obstructive sleep apnea syndrome. Additionally, vitamin B12, vitamin D, folic acid, and thyroid‐stimulating hormone levels were detected in lab investigations and analyzed as continuous variables. Finally, medication evaluation was based on the use of medications identified by the Beers criteria as having potential negative implications for cognitive health in dementia or cognitive decline (American Geriatrics Society Beers Criteria® Update Expert Panel, 2019).

In this study, the Fazekas scale was employed to evaluate microvascular ischemic changes within the brain's white matter. Specifically, the deep white matter component of the scale, referred to as FAZEKAS DWM, assesses the extent of abnormal white matter signal intensity in the supratentorial regions. This assessment utilizes T2‐weighted or FLAIR MRI sequences, with abnormalities rated on a scale ranging from 0 to 3. A score of 0 signifies no evidence of white matter lesions, 1 indicates isolated focal lesions, 2 is assigned for early confluent lesions, and a score of 3 denotes widespread involvement of the white matter with diffuse lesions (Fazekas et al., 1987).

### 2.6 Neuropsychological evaluation

For the neuropsychological evaluation, a variety of NPTs were employed to assess different cognitive domains. These included the Oktem Auditory Verbal Learning Test (for memory domain), Forward and Backward Digit Span (for simple and complex attention), Trail Making Test (TMT) (for executive functioning and attention), Stroop (for psychomotor speed and executive functions), Phonemic and Semantic Fluency, and Clock Drawing (CDT) (visuospatial) tests were used. All tests were standardized for Turkish; *z*‐scores were used (Cangöz et al., 2006, 2013; Karakaş et al., 1999; Sezgi̇ et al., 2017; Tanör, 2011). Psinorm was used to calculate the norm values and organize the neuropsychology database (Ayhan et al., 2022). For participants with lower levels of education, the TMT, Stroop Test, phonemic fluency, and CDT were not used due to the absence of appropriate normative studies for these subgroups. A participant's performance was considered abnormal if they exhibited a deficit of more than 1.5 SD on any single NPT described above or a deficit of more than 1.0 SD on two tests within the same cognitive domain or across three different domains (Molinuevo et al., 2017).

### 2.7 Magnetic resonance imaging

MRI examinations were performed on 1.5 T MRI (Siemens Symphony Tim 1.5 T MRI, Siemens Medical Solutions) utilizing an ultrafast gradient‐echo 3D sequence (MPRAGE) 3D T1 weighted magnetization prepared rapid gradient echo (MPRAGE) sequence (TR = 1800 ms, TE = 3.57 ms, FA = 15, matrix size = 192 × 256; slice thickness = 1.0 mm; FOV = 180 × 240 mm.), which was used to measure cortical volume globally and in specific cortical regions corresponding with the tissue samples extracted.

Cortical reconstruction and volumetric segmentation were performed with the Freesurfer image analysis suite v6.0, which is documented and freely available for download online (http://surfer.nmr.mgh.harvard.edu/). The technical details of these procedures are described in prior publications (Dale & Sereno, 1993; Dale et al., 1999; Desikan et al., 2006; Fischl & Dale, 2000; Fischl et al., 2001, 2002, 2004; Fischl, Sereno et al., 1999; Fischl, Sereno, & Dale 1999; Han et al., 2006; Jovicich et al., 2006; Reuter et al., 2010, 2012; Ségonne et al., 2004; Sled et al., 1998). Procedures for measuring cortical thickness have been validated against histological analysis (Rosas et al., 2002) and manual measurements (Kuperberg et al., 2003; Salat et al., 2004). Freesurfer morphometric procedures have shown good test–retest reliability across scanner manufacturers and across field strengths (Han et al., 2006; Reuter et al., 2012).

### 2.8 Meta‐ROIs

Based on the literature review, we have decided to use three different cortical thicknesses and two cortical volume patterns compatible with the different stages of AD. The first meta‐ROI, the AD cortical signature (AD‐CS) (Dickerson et al., 2009), comprises nine cortical areas (Dickerson & Wolk, 2012; Dickerson et al., 2011). In this study, the AD‐CS included the following regions: (1) middle temporal cortex, (2) inferior temporal cortex, (3) temporal pole, (4) superior frontal cortex, (5) superior parietal cortex, (6) supramarginal gyrus, (7) precuneus, (8) middle frontal cortex, and (9) angular gyrus (AG). We were not able to include AG in our study since the Desikan–Killiany atlas that we use in our Freesurfer analyses did not include AG (Desikan et al., 2006).

Next, a progression predictive cortical thickness (PP‐CT) was obtained by taking the average cortical thickness of 4 regions which predicted the progression to AD, in MCI: (1) middle temporal cortex, (2) inferior temporal cortex, (3) temporal pole, and (4) superior frontal cortex (Bakkour et al., 2009).

Third, the familial AD cortical signature (fAD‐CS), which consisted of six ROI's that differed the mildest symptomatic mutation carriers, was evaluated: (1) entorhinal cortex, (2) inferior parietal cortex, (3) precuneus, (4) superior frontal cortex, (5) superior parietal cortex, and (6) supramarginal gyrus (Weston et al., 2016).

As for the volumes, the discriminant gray matter volumes for AD (DGMV‐AD) were calculated, which had the highest discrimination value between healthy controls and AD: (1) hippocampus, (2) entorhinal cortex, (3) amygdala, (4) middle temporal cortex, (5) inferior temporal cortex, and (6) temporal pole (Schwarz et al., 2016).

**FIGURE 1:**
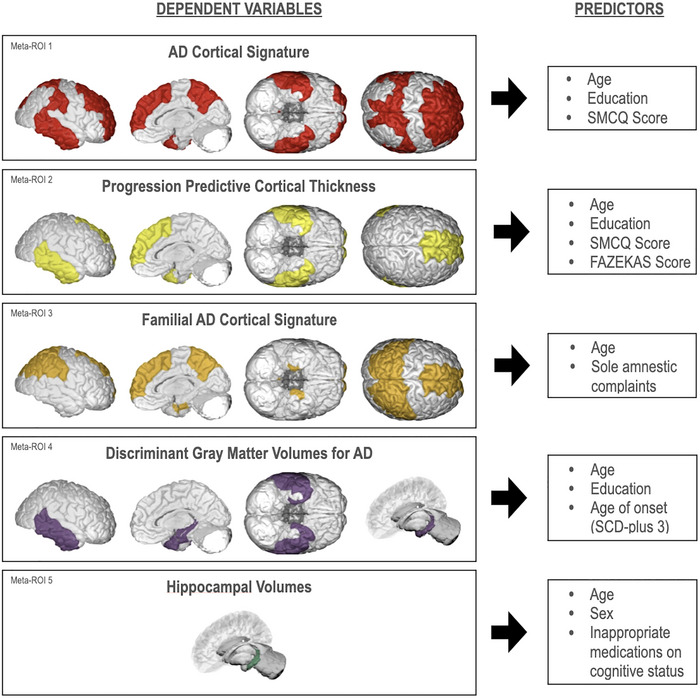
This figure displays the selected five meta‐ROIs derived from literature review, which exhibit patterns of cortical thinning and volume reduction associated with various stages of Alzheimer's disease. A regression analysis revealed clinical variables predictive of atrophy within these meta‐ROIs in subjective cognitive decline (SCD) sample, as displayed on the right.

Lastly, the hippocampus volumes were also measured (Fan et al., 2018; Ryu et al., 2017; Yue et al., 2018). The ROIs used in this study can be seen in Figure 1, and measured mean values can be seen in Tables S3 and 4.

The following laterality index (LI) was used for both thickness and volume to adjust the asymmetry (Kong et al., 2022):

LI=100×(QLHQRH)/(QLH+QRH)

(QLH and QRH are representative cortical thickness or regional volume measurements for the LH and RH contributions, respectively).

The raw volumes were corrected for ICV by dividing each structure's raw volume by TIV for each individual.

### 2.9 Statistical analysis

Statistical analyses were performed using R version 4.0.3 for macOS. Out of all the data evaluated, the missing values across the independent variables varied between 0% and 32%. In total, 1601 out of 3328 records (5.5%) were incomplete. Therefore, we used multiple imputation to create and analyze 200 multiply imputed datasets. Incomplete variables were imputed under fully conditional specification, using the default settings of the mice 3.13.0 package (Van Buuren & Groothuis‐Oudshoorn, 2011).

The following variables were considered in the study: Sex, SCD‐plus criterion 3, 4, 5, 6, complaints related to non‐amnestic domains, using medications that have negative implications for cognitive health (according to Beers criteria), and NPTs for evaluating cognitive function were treated as binomial variables. Family history of neurocognitive disorders, FAZEKAS DWM scores, age, education, meta‐ROI scores, lateralization indexes, SCD‐plus 2, SMCQ total scores, 3MS z scores, comorbidity scores, vitamin B12, vitamin D, folic acid, and TSH levels were regarded as continuous variables.

To select variables for the regression analyses, we performed stepwise model selection on each imputed dataset separately. After that, we created new supermodels for each dependent variable (meta‐ROIs), which included all variables present in at least half of the original models. Then, we applied a backward elimination procedure to all variables in the supermodel. We calculated the Wald test *p*‐value by removing each variable from the model in turn. If the *p*‐value exceeds.05, we remove the corresponding variable and repeat the procedure with the remaining model. If all *p*≤.05, we stopped the procedure. The parameters of supermodels were estimated in each imputed dataset separately and combined using Rubin's rules. This method is reliably used in the presence of missing data and is preferred over listwise deletion (White & Carlin, 2010). To present the results gathered from the unaltered dataset, we also conducted a listwise deletion, repeated the regression procedure described above, and presented the results in the Supporting Information.

## 3 RESULTS

### 3.1 The descriptives

A total of 104 individuals were evaluated, with an average age of 67.6 years (Range = 55–91). The majority of the participants were female (69.2%), and the average education was low (8.4 ± 5.1). About one‐quarter of the participants (26.2%) reported having a family history of major neurocognitive disorders.

During the evaluation, 21.2% of the sample were diagnosed with a mental disorder (depressive or anxiety disorder). Vascular risk factors were high among participants, the most common being hypertension (46.2%) followed by Type 2 diabetes 29.8%, hyperlipidemia in 28.8%, and almost a quarter had a history of coronary artery disease. Overall, 10.6% of participants were taking medications that could affect their cognitive status according to Beers criteria. The median vitamin D level was 21.25 µg/L. Participant demographics and clinical characteristics are shown in Table 1.

**TABLE 1:** Demographic and clinical features of the study population.

Demographic features	
**Age** (year, mean, SD)	67.6 (8.2)
**Sex** (Female, *n*, %)	72 (69.2)
**Education** (year, mean, SD)	8.4 (5.1)
**Clinical features**	
**Non‐amnestic complaints** (*n*, %)	48 (46.2)
**SMCQ total score** (mean, SD)	6.3 ± 2.5
**Cognitive decline according to detailed neuropsychological testing** (*n*, %)	7 (6.7)
**Family history of NCDs** (*n*, %)	
**None**	76 (73.1)
**1st degree relatives**	17 (16.3)
**2nd degree relatives**	7 (6.7)
**3rd degree relatives**	3 (2.9)
**Comorbidities** (*n*, %)	
**Depressive or anxiety disorder**	22 (21.2)
**Coronary artery disease**	24 (23.1)
**Diabetes mellitus Type 2**	31 (29.8)
**Hyperlipidemia**	30 (28.8)
**Hypertension**	48 (46.2)
**Cerebrovascular accident**	2 (1.9)
**Migraine**	8 (7.7)
**Head trauma**	5 (4.8)
**Chronic kidney failure**	2 (1.9)
**Obstructive sleep apnea syndrome**	2 (1.9)
**Medications impacting cognitive health negatively** (*n*, %)	11 (10.6)
**Vitamin B12** (ng/mL, mean, SD)	298.40 (133.69)
**Folic acid** (µg/L, mean, SD)	10.49 (3.90)
**Vitamin D** (µg/L, median, IQR)	21.25 (18.98)
**TSH** (IU/mL, median, IQR)	1.65 (1.59)

Abbreviations: NCDs, neurocognitive disorders; SCD, subjective cognitive decline; SD, standard deviation; SMCQ, subjective memory complaints questionnaire; TSH, thyroid stimulating hormone.

Out of the total sample, 83.7% reported cognitive complaints in the past 5 years (SCD‐plus 2), and 63.5% experienced SCD onset after the age of 60 (SCD‐plus 3) (Table 2). A total of 80 participants (76.9%) expressed concerns about their cognition (SCD‐plus 4). In comparison to others in their age group, 38.5% of participants believed their cognitive performance was worse (SCD‐plus 5). Sixty‐nine participants had informants who were available and consented to participate in the study. Within this subsample, 53 informants (76.8% of the subsample with informants) confirmed the participants’ reports of cognitive decline, satisfying the SCD‐plus 6 criterion. In total, 91.3% met at least one SCD‐plus criteria, with an average of 3.1 criteria met per participant. Only 12.5% of participants fulfilled all SCD criteria.

**TABLE 2:** Subjective cognitive decline (SCD)‐plus status of the study population.

SCD‐plus status	
**SCD‐plus 2**: Onset of SCD within the last 5 years (*n*, %)	87(83.7)
**SCD‐plus 3**: Age at onset of SCD is over the 60 years (*n*, %)	66 (63.5)
**SCD‐plus 4**: Concerns associated with SCD (*n*, %)	80 (76.9)
**SCD‐plus 5**: Feeling of worse performance than others of the same age group (*n*, %)	30 (38.5)
**SCD‐plus 6**: Confirmation of cognitive decline by an informant (*n*, %)	53 (51.0)
**Number of participants with at least one SCD‐plus criteria** (*n*, %)	95 (91.3)
**Number of SCD‐plus criteria per individual** (mean, SD)	3.1 (1.4)
**Number of participants fulfilling all SCD criteria** (*n*, %)	13 (12.5)

### 3.2 Factors that associated with AD‐related atrophy in SCD

For each of the five meta‐ROIs, we created supermodels to determine which independent variables predicted them (Figure 1). Therefore, the findings that will be presented henceforth have been controlled for all the independent variables of the research.

In the stepwise model selection process, the detailed neuropsychological evaluation with NPTs, family history, and comorbidity scores were not chosen for any of the dependent variables in at least half of the imputed datasets. Consequently, it was not included in the supermodels.

For clarity, we will present the independent variables that predict meta‐ROIs in an order aligned with our research questions.

#### 3.2.1 Demographic factors

**FIGURE 2:**
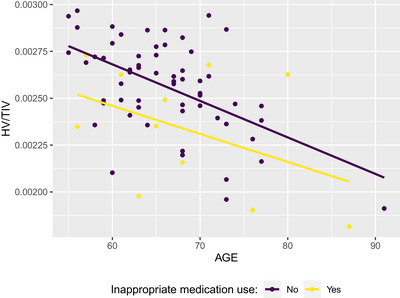
This graph displays the relationship between age and hippocampal volume index corrected for total intracranial volume (HV/TIV) in female participants. Yellow dots indicate individuals who have been prescribed medications that have negative implications for cognitive health, according to Beers criteria. In contrast, purple dots denote individuals without such medication use. Linear regression lines illustrate a more pronounced decline in HV/TIV with age among those on medications that have negative implications for cognitive health, suggesting a potential impact on hippocampal volume.

**FIGURE 3:**
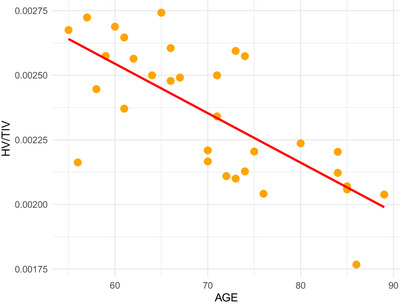
The scatter plot delineates the inverse correlation between age and HV/TIV in male subjects with subjective cognitive decline (SCD). The red regression line demonstrates this negative trend, indicating age‐associated hippocampal atrophy in the male SCD cohort. Notably, the graph does not display male participants prescribed medications that have negative implications for cognitive health, as no cases were present in the dataset.

After the imputation and the regression analyses of supermodels, lower scores in all meta‐ROIs were associated with increased age (Table 3). Sex may be a predictor of hippocampal volume in individuals with SCD; men with SCD had smaller hippocampal volumes than women (B = −.013, *p* = .005) (for visual representation refer to Figures 2 and 3). Higher education was associated with thinner cortices in AD‐CS (B = −.014, *p* = .019), PP‐CT (B = −.020, *p* = .003), and smaller volumes in DGMV regions (B = −.014, *p* = .025).

**TABLE 3:** Supermodels created by variable selection and results of regression analysis (with multiple imputed data, *m* = 200).

		*B*	SE	*t*‐Statistic	df	*p* Value	Confidence interval		*R* ^2^	Adj‐*R* ^2^
							2.5%	97.5%		
**AD‐CS**	**Age**	−.026	.004	−7.068	96.092	.000	.967	.981	.452	.430
	**Education**	−.014	.006	−2.392	94.313	.019	.974	.998		
	**LI**	.089	.028	3.177	95.249	.002	1.034	1.155		
	**SMCQ score**	−.031	.014	−2.227	74.718	.029	.944	.997		
**PP‐CT**	**Age**	−.024	.005	−5.368	94.674	.000	.967	.985	.480	.453
	**Education**	−.020	.007	−3.026	92.971	.003	.967	.993		
	**LI**	.067	.018	3.703	94.540	.000	1.031	1.108		
	**SMCQ score**	−.037	.015	−2.392	70.766	.019	.935	.994		
	**FAZEKAS DWM**	−.095	.045	−2.091	92.487	.039	.831	.995		
**fAD‐CS**	**Age**	−.023	.003	−6.961	98.475	.000	.971	.984	.361	.349
	**Non‐amnestic complaints**	.118	.057	2.074	88.410	.041	1.005	1.260		
**DGMV**	**Age**	−.033	.005	−6.400	88.852	.000	.958	.977	.358	.339
	**Education**	−.014	.006	−2.272	97.084	.025	.975	.998		
	**SCD‐plus 3: age of onset**	−.262	.094	−2.798	83.754	.006	1.079	1.565		
**HV**	**Age**	−.002	.000	−7.305	97.753	.000	.998	.999	.446	.430
	**Sex**	.013	.005	2.851	97.705	.005	1.004	1.023		
	**Medications impacting cognitive health negatively**	−.018	.007	−2.692	97.891	.008	.969	.995		

Abbreviations: AD‐CS, Alzheimer's disease cortical signature; B, unstandardized regression coefficient; df, degree of freedom; DGMV‐AD, discriminant gray matter volumes for Alzheimer's disease; fAD‐CS, familial Alzheimer's disease cortical signature; LI, laterality index; PP‐CT, progression predictive cortical thickness; SCD, subjective cognitive decline; SE, standard error; SMCQ, subjective memory complaints questionnaire.

#### 3.2.2 SMCQ

Next, we evaluated whether using SMCQ would add to the clinical evaluation in people with cognitive complaints. Higher scores in SMCQ were associated with thinner cortices in AD‐CS and PP‐CT (B = −.031, *p* = .029 and B = −.037, *p* = .019). Asking whether the complaints are constrained in memory helped to identify morphological alterations; in participants with sole amnestic complaints, the fAD‐CS thickness was reduced (B = −.118, *p* = .041). Our analysis did not differentiate between the specific types of non‐amnestic complaints in relation to structural changes, as these complaints were examined collectively under the “Non‐amnestic complaints” variable.

#### 3.2.3 SCD plus criteria

When SCD‐plus criteria were assessed, a reported age of onset at 60 years or older was associated with smaller gray matter volumes in AD, when adjusted for the current age (B = −.26, *p* = .006). No significant correlations were found for other SCD‐plus criteria.

#### 3.2.4 Exclusionary criteria

Using drugs with adverse cognitive effects was significantly associated with small hippocampi (B = −.018, *p* = .008) (illustrated in Figure 2). In our analyses for the effect of deep white matter lesions, thinner cortices in PP‐CT were associated with increased FAZEKAS deep white matter scores (B = −.095, *p* = .039).

## 4 DISCUSSION

This study examined how different SCD criteria are associated with morphological changes related to AD. In the presence of subjective cognitive complaints, AD‐related morphological changes were associated with advanced age, male sex, higher education, later age of onset, complaints restricted to the memory domain, deep white matter vascular burden, and using medications that have negative implications for cognitive health when subjective cognitive complaints were present. Higher scores in a standardized survey were also associated with reduced volumes. Assessing the SCD‐plus criteria, conducting a comprehensive neuropsychological evaluation, and evaluating a family history of neurocognitive disorders and comorbidities did not help to detect morphological changes.

We conducted an in‐depth study on the SCD concept with respect to the markers of degeneration in AD and all relevant clinical information. Our analysis included psychiatric and medical comorbidities, first‐line lab results, and medication use that could impact cognitive performance. This information is available to clinicians worldwide in daily settings, reflecting real‐life situations. Moreover, we did not limit our analysis to the cortical signature of AD. We examined five morphological meta‐ROIs suggested in the literature, including three different cortical thicknesses and two regional brain volume composite scores.

Yet, it is important to acknowledge the existence of various distinctive subtypes of brain atrophy in AD, beyond the meta‐ROIs we examined. However, the typical pattern of brain atrophy in AD involves the hippocampus and association cortices (including lateral parietal, temporal, and frontal regions) the literature also describes distinct clinical and imaging subtypes (Ferreira et al., 2017; Lam et al., 2013). However, as the scope of the study was to evaluate the predictive clinical factors for AD‐specific atrophy patterns in the SCD population, we limited our analysis to typical and more commonly observed atrophy patterns in AD. Research designs involving a large number of participants and control groups can a priori evaluate regional differences between individuals with SCD and cognitively healthy individuals, thereby facilitating the investigation of the presence of other subtypes and the clinical factors that predict them.

Below, we will discuss the factors that are associated with AD‐related morphological changes.

*Age*: The effect of advanced age on the risk of atrophy in AD‐associated brain regions was consistent for all meta‐ROIs. The impact of age on the risk of AD is well‐established. Older individuals with SCD have been shown to carry a higher risk of progression to amnestic MCI (Rabin et al., 2020).

*Sex*: Studies have previously suggested that women with SCD are at a higher risk of cognitive decline and dementia (Heser et al., 2019; Oliver et al., 2022). Interestingly, in our study, men with SCD had smaller hippocampal volumes. This observation could be attributed to the earlier findings that men may require more pathological changes to exhibit similar cognitive symptoms as women (Barnes et al., 2005).

Another possible explanation could be related to the assessment method used. We normalized the raw volumes of each ROI by dividing them with the ICV. However, previous research has found that males had greater raw volumes in many other brain structures, except for the hippocampus (Barnes et al., 2010; Voevodskaya et al., 2014). Our data also showed no significant difference in hippocampal volumes between sexes (Table S7). As males had a higher mean ICV, there might have been a bias toward smaller hippocampal volumes in males only.

To avoid this bias, raw volumes without normalization could be used. However, it would not be possible to adjust the brain estimates for interindividual differences in head size. Another normalization method that can be applied is using the residuals of a least‐square‐derived linear regression between raw volumes and ICV (Voevodskaya et al., 2014). Yet, this method would remove any volumetric sex dimorphism (with all other ICV‐related variance), leading to an underestimation of true differences in males and females (Mathalon et al., 1993).

*Education*: Higher education was associated with atrophy in regions of three different meta‐ROIs. This observation aligns with prior findings indicating an increased risk of AD in individuals with higher education who report subjective complaints (Jonker et al., 2000; Van Oijen et al., 2007). Aghjayan et al. (2017) displayed that the link between SCD and amyloid positivity strengthens with higher levels of education. These results suggest either that individuals with more years of education exhibit a unique vulnerability or merely a detection bias due to their greater cognitive reserve (Ge et al., 2019; Livingston et al., 2020; Strenze, 2007; Van Hootegem et al., 2023). Therefore, although older individuals with higher education are potentially more adept at compensating for symptoms associated with cognitive decline in their daily lives, this compensatory ability might also mask underlying pathologies, leading to a later presentation when the disease has progressed further. These findings differ from Janssen et al. (2022), whose study discovered that a higher education was correlated with a lower frequency of amyloid positivity in individuals with SCD, suggest that the role of education in the context of amyloid pathology and SCD may be multifaceted and warrants further investigation.

*Assessment methods of cognitive complaints*: AD‐like cortical thinning pattern was detected in individuals with higher scores at SMCQ (higher cognitive complaints). This finding is in line with the previous studies (Dauphinot et al., 2020; Kuhn et al., 2019), suggesting that it would be helpful to investigate the characteristics and rate the severity of complaints in SCD in clinics (Diaz‐Galvan et al., 2021).

Our study detected an AD‐like cortical thinning pattern in individuals who reported sole amnestic complaints. Although the initial symptoms of AD may relate to various cognitive domains, existing literature suggests that preclinical AD is primarily linked to memory complaints. Our findings are consistent with recent research by Janssen et al. (2022), which revealed that individuals with complaints specifically related to memory had a higher incidence of amyloid positivity (Janssen et al., 2022).

*SCD‐plus criteria*: We examined the effectiveness of using SCD‐plus criteria to determine AD‐related atrophy. Only a later age of onset was linked to a decrease in DGMV‐AD, after controlling for the age at participation. As the risk of AD increases with age, late‐onset subjective cognitive complaints may be more connected to preclinical AD. These findings align with recent findings, where only age of onset and APOE ε4 status were significantly associated with AD‐related amyloid or tau deposition (Janssen, et al., 2022; Slot et al., 2016).

*Neuropsychological evaluation*: Our study found that using NPT did not provide any additional predictive power to determine morphology. This negative finding could be attributed to the good psychometric properties of 3MS in our study population. However, the outcome may also have been influenced by the sample size. We observed that 6.7% of the participants displayed deficits in thorough neuropsychological evaluation. By definition, SCD is characterized by cognitive complaints in the absence of objective decline on NPT. In our study, SCDs were based on the presence of complaints despite the normal performance on the cognitive screening test. This approach was chosen to explore the value of detailed NPT over conventional cognitive screening in predicting AD‐related atrophy patterns.

*Vascular burden*: We found that vascular burden was associated with AD‐like cortical thinning pattern. The median FAZEKAS score was 1, indicating a significant vascular burden in our study population. It is widely accepted that factors that impaired vascular health can significantly contribute to the development of AD (Chui et al., 2006). There is evidence that vascular deterioration and amyloid deposition affect each other in both directions (Iadecola & Gottesman, 2018), and it is suggested that vascular burden is associated with increased AD‐related atrophy patterns in pre‐dementia stages 2013 (Guzman et al., 2013). However, we did not utilize a conventional risk score such as Framingham, which could have provided additional data on vascular contribution.

*Using medications that have negative implications for cognitive health*: Our study revealed that medications impacting cognitive health negatively according to Beers criteria can lead to a decrease in hippocampal volume, which is in line with previous studies (Risacher et al., 2016; Weigand et al., 2020). Anticholinergic drugs can potentially promote the development of AD pathology (Wurtman, 2015).

*Comorbidities*: SCD‐I recommends excluding comorbidities that may cause cognitive complaints in order to improve the predictive power of SCD. We did not observe any effect of anxiety and depression on structural biomarkers. All participants were evaluated by a psychiatrist, and the diagnoses were coded as binary variables. We did not use a standardized scale, which prevented us from analyzing the symptoms as continuous variables (Parmelee et al., 1989). Such an approach might have limited the assessment of the effects of subthreshold clinical symptoms.

*Limitations*: It is important to note that in this study, we did not have access to information on the participants’ amyloid and tau markers or their APOE ε4 status. These pathophysiologic biomarkers, despite their crucial role in AD progression, are not yet widely used globally due to various constraints, including resource limitations. Instead, we relied on widely available clinical methods to identify certain predictors of AD‐related atrophy in individuals with SCD.

This approach allowed us to explore the potential of non‐biomarker criteria within the SCD‐plus framework to predict AD‐related structural changes. However, to better validate the SCD criteria and fully understand the impact of these pathophysiologic biomarkers on SCD progression, a replication study incorporating amyloid and tau status, as well as APOE ε4 genotype information and follow‐up data, would be necessary. Such studies would provide a more comprehensive understanding of the mechanisms underlying SCD and its progression to AD, underscoring the importance of integrating these biomarkers into future research.

## 5 CONCLUSION

In this study, we concluded that individuals with subjective complaints who are at an advanced age, higher education, male sex, later age of onset of cognitive complaints, and using medications that have negative implications for cognitive health may display smaller AD‐related structures. The chance of catching the difference increases with a thorough cognitive evaluation. Future studies should focus on which clinical features better predict AD‐related pathological changes in SCD while using multiple biomarker modalities in a longitudinal design.

## AUTHOR CONTRIBUTIONS

**Ahmet Alp Karakasli**: Conceptualization; investigation; funding acquisition; writing—original draft; methodology; visualization; writing—review and editing; software; formal analysis; data curation; validation. **Esra Ozkan**: Data curation; resources; investigation. **Duygu Cap**: Investigation; software; data curation. **Melike Karacam Dogan**: Investigation; data curation. **Karaosmanoglu**: Investigation; software; resources; supervision. **Sevilay Karahan**: Formal analysis; data curation; supervision; writing—review and editing. **Nabi Zorlu**: Supervision; formal analysis; software; methodology; writing—review and editing. **Esen Saka**: Conceptualization; investigation; supervision; writing—review and editing. **Yavuz Ayhan**: Conceptualization; investigation; funding acquisition; writing—original draft; methodology; validation; visualization; writing—review and editing; software; formal analysis; project administration; data curation; supervision; resources.

## CONFLICT OF INTEREST STATEMENT

All authors certify that they have no affiliations with or involvement in any organization or entity with any financial interest (such as honoraria; educational grants; participation in speakers’ bureaus; membership, employment, consultancies, stock ownership, or other equity interest; and expert testimony or patent‐licensing arrangements) or nonfinancial interest (such as personal or professional relationships, affiliations, knowledge, or beliefs) in the subject matter or materials discussed in this manuscript.

## FUNDING INFORMATION

Scientific and Technological Research Council of Turkey,Grant Number: 214S048; Psychiatric Association of Turkey, and HU Scientific Research Projects Coordination Unit, Grant Number: THD 2018‐17363

### PEER REVIEW

The peer review history for this article is available at https://publons.com/publon/10.1002/brb3.3506.

## Supporting information

Supporting Information

## Data Availability

The data supporting this study's findings are available from the corresponding author, YA, upon reasonable request.
