# Supplementary material for: Clinical predictors of Alzheimer's disease‐like brain atrophy in individuals with memory complaints
Source: Brain Behav. 2024 Apr 30;14(5):e3506. doi: 10.1002/brb3.3506 (PMC11061206; doi:10.1002/brb3.3506)
Supplement: Supplementary file 1 — Supporting Information [file BRB3-14-e3506-s001.docx]

**Supp. Table 1.** The questions created by investigators to collect information on non-amnestic cognitive domains

| **Questions** | **Cognitive Domain** |
| --- | --- |
| Do you have difficulties… |  |
| finding the right words to use in a conversation? | Language |
| telling someone else about a program watched on TV? |  |
| remembering the meaning of frequently used words? |  |
| understanding what others are trying to say? |  |
| following a map to find a place? | Visuospatial |
| giving directions to someone by looking at the map? |  |
| finding your way through a familiar neighborhood? |  |
| doing two jobs at the same time? | Attention |
| concentrating on a task without being distracted by external stimuli? |  |

**Supp. Table 2.** Correlations between meta-ROIs identified as dependent variables in the study

|  | **AD-CS** | **PP-CT** | **fAD-CS** | **Proportionally normalized DGMV-AD** | **Proportionally normalized HV** |
| --- | --- | --- | --- | --- | --- |
| **AD-CS** | 1.000 |  |  |  |  |
| **PP-CT** | 0.932^*^ | 1.000 |  |  |  |
| **fAD-CS** | 0.917^*^ | 0.805^*^ | 1.000 |  |  |
| **DGMV** | 0.545^*^ | 0.571^*^ | 0.507^*^ | 1.000 |  |
| **HV** | 0.527^*^ | 0.540^*^ | 0.514^*^ | 0.746^*^ | 1.000 |

Abbreviations: AD-CS, Alzheimer’s disease cortical signature; PP-CT, progression predictive cortical thickness; fAD-CS, familial Alzheimer’s disease cortical signature; DGMV, discriminant grey matter volumes for Alzheimer’s disease.

* Statistically significant for correlation, p< 0.01.

**Supp. Table 3.** Cortical thicknesses (mm) used in meta-ROIs

| **ROIs** | **Right** | | **Left** | |
| --- | --- | --- | --- | --- |
|  | **Mean** | **SD** | **Mean** | **SD** |
| **Entorhinal** | 3.385 | 0.388 | 3.233 | 0.369 |
| Middle temporal | 2.797 | 0.191 | 2.769 | 0.226 |
| Inferior temporal | 2.840 | 0.228 | 2.794 | 0.239 |
| Temporal pole | 3.586 | 0.410 | 3.486 | 0.463 |
| Superior frontal | 2.642 | 0.193 | 2.649 | 0.198 |
| Middle frontal | 4.657 | 0.298 | 4.867 | 0.333 |
| Superior parietal | 2.097 | 0.171 | 2.136 | 0.169 |
| Inferior parietal | 2.400 | 0.176 | 2.409 | 0.194 |
| Supramarginal | 2.389 | 0.156 | 2.396 | 0.174 |
| Precuneus | 2.238 | 0.173 | 2.217 | 0.170 |

Abbreviations: SD, standard deviation.

**Supp. Table 4.** Regional brain volumes (mm^3^) used in meta-ROIs

| **ROIs** | **Right** | | **Left** | |
| --- | --- | --- | --- | --- |
|  | **Mean** | **SD** | **Mean** | **SD** |
| **Hippocampus** | 3112.7 | 398.4 | 3015.5 | 377.6 |
| **Entorhinal** | 1957.9 | 408.5 | 2001.2 | 435.0 |
| Amygdala | 1485.6 | 234.9 | 1282.9 | 211.2 |
| Middle temporal | 10304.1 | 1265.2 | 9589.9 | 1305.7 |
| Inferior temporal | 10029.1 | 1343.4 | 10584.1 | 1517.4 |
| Temporal pole | 2383.2 | 414.5 | 2302.7 | 453.7 |

Abbreviations: SD, standard deviation.

NOTE. The mean estimated total intracranial volume was 1447654.9, and standard deviation was 138852.1.

**NOTE**. For analysis with the data of only completed cases, univariate analysis were used for variable selection. Correlation was used for continuous variables, and student’s t-test for categorical variables for univariates analyses. A significance level of *p* < 0.25 were used as a threshold for linear regression models. Additionally, age, gender, education and laterality index were used as constant variables. Second, a stepwise model was used. A p-value of < 0.05 was used for statistical significance.

**Supp Table 5.** Univariate analysis for the predictors of metaROIs

|  | **AD-CS** | **fAD-CS** | **PP-CT** | **DGMV-AD** | **HV** |
| --- | --- | --- | --- | --- | --- |
| **SMCQ total scores** | 0.830 | 0.485 | 0.828 | 0.438 | 0.512 |
| **SCD-plus 2:** Onset of SCD | 0.838 | 0.827 | 0.685 | 0.98 | 0.726 |
| **SCD-plus 3:** AOO > 60 y | 0.002* | 0.000* | 0.001* | 0.169* | 0.003* |
| **SCD-plus 4:** Concerns of SCD | 0.028* | 0.033* | 0.067* | 0.073* | 0.355 |
| **SCD-plus 5:** Feeling of worse than the same age group | 0.455 | 0.09* | 0.781 | 0.835 | 0.562 |
| **SCD-plus 6:** Confirmation by an informant | 0.355 | 0.288 | 0.171* | 0.392 | 0.416 |
| **Non-amnestic complaints** | 0.173* | 0.098* | 0.399 | 0.268 | 0.063* |
| **Family history of NCDs** | 0.348 | 0.211* | 0.561 | 0.554 | 0.154* |
| **Psychiatric comorbidities (depression and anxiety disorders)** | 0.156* | 0.053* | 0.197* | 0.962 | 0.55 |
| **Vascular comorbidities** | 0.229* | 0.361 | 0.129* | 0.628 | 0.898 |
| **Other comorbidities** | 0.918 | 0.827 | 0.864 | 0.209* | 0.93 |
| **Vitamin B12 levels** | 0.386 | 0.394 | 0.175* | 0.048* | 0.438 |
| **Vitamin D levels** | 0.849 | 0.971 | 0.67 | 0.180* | 0.586 |
| **Folic acid levels** | 0.845 | 0.749 | 0.435 | 0.839 | 0.595 |
| **TSH levels** | 0.446 | 0.689 | 0.479 | 0.819 | 0.761 |
| **Using inappropriate medications on cognitive status** | 0.480 | 0.313 | 0.767 | 0.166* | 0.169* |
| **3MS z scores** | 0.732 | 0.644 | 0.505 | 0.653 | 0.396 |
| **Using NPTs** | 0.023* | 0.025* | 0.021* | 0.121* | 0.098* |
| **FAZEKAS scores** | 0.004* | 0.033* | 0.001* | 0.000* | 0.001* |
| **LI** | 0.006* | 0.551 | 0.021* | 0.106* | 0.138* |

**Supp Table 6.** Stepwise regression analysis restricted the completed cases only for predicting the meta-ROI scores .

|  |  | **Unstandardized Coefficients** | | **Standardized Coefficients** | |  |  |  |
| --- | --- | --- | --- | --- | --- | --- | --- | --- |
|  |  | **B** | **SE** | **β** | **Sig.** | **R^2^** | **F** | **p** |
| **AD-CS** (N=82) | **Age** | -0.023 | 0.004 | -0.502 | 0.000 | 0.374 | 25.173 | 0.000 |
|  | **LI** | 0.099 | 0.031 | 0.283 | 0.002 |  |  |  |
| **PP-CT** (N=60) | **Age** | -0.028 | 0.006 | -0.500 | 0.000 | 0.427 | 15.629 | 0.000 |
|  | **LI** | 0.079 | 0.027 | 0.288 | 0.006 |  |  |  |
|  | **Education** | -0.021 | 0.010 | -0.217 | 0.034 |  |  |  |
| **fAD-CS** (N=77) | **Age** | -0.022 | 0.004 | -0.57 | 0.000 | 0.316 | 36.075 | 0.000 |
| **DGMV-AD** (N=66) | **Age** | -0.032 | 0.005 | -0.761 | 0.000 | 0.552 | 20.333 | 0.000 |
|  | **LI** | 0.043 | 0.013 | 0.283 | 0.001 |  |  |  |
|  | **SCD-plus 3:** AOO > 60 y | -0.321 | 0.090 | -0.403 | 0.001 |  |  |  |
|  | **Using NPTs** | -0.137 | 0.066 | -0.184 | 0.042 |  |  |  |
| **HV** (N=82) | **Age** | -0.002 | 0.000 | -0.619 | 0.000 | 0.489 | 26.786 | 0.000 |
|  | **Using inappropriate medications on cognitive status** | -0.024 | 0.007 | -0.275 | 0.001 |  |  |  |
|  | **Gender** (Female) | 0.012 | 0.005 | 0.189 | 0.026 |  |  |  |

Abbreviations: AD-CS, Alzheimer’s disease cortical signature; PP-CT, progression predictive cortical thickness; fAD-CS, familial Alzheimer’s disease cortical signature; DGMV-AD, discriminant grey matter volumes for Alzheimer’s disease; Prop., proportionally normalized; AOO, age of onset; NPTs, neuropsychological tests; LI, laterality index.

**Supp Table 7.** Hippocampal volumes between genders

|  | **Males** | | **Females** | | **t(101)** | ***p*** | **Cohen’s *d*** |
| --- | --- | --- | --- | --- | --- | --- | --- |
|  | **M** | **SD** | **M** | **SD** |  |  |  |
| **Hippocampal volume** | 3670.02 | 464.91 | 3517.15 | 444.98 | 1.59 | 0.05 | 0.34 |
